# A Rare Case of Solitary Primary and Recurrent Hepatic Epithelioid Hemangioendothelioma Undergoing Repeat Liver Resections

**DOI:** 10.70352/scrj.cr.24-0084

**Published:** 2025-04-17

**Authors:** Yuhi Yoshizaki, Fuyuki Inagaki, Mai Nakamura, Takashi Kokudo, Fuminori Mihara, Nobuyuki Takemura, Norihiro Kokudo

**Affiliations:** Hepato-Biliary-Pancreatic Surgery Division, Department of Surgery, National Center for Global Health and Medicine, Tokyo, Japan

**Keywords:** hepatic epithelioid hemangioendothelioma, recurrence, liver resection

## Abstract

**INTRODUCTION:**

Hepatic epithelioid hemangioendothelioma (HEHE) is a rare vascular tumor. Treatment strategy remains controversial because of its rarity. Liver resection is considered as the optimal treatment for solitary HEHE, while a small subset of patients have a solitary tumor. We present the rare case of a patient with solitary primary HEHE who experienced solitary recurrence following liver resection and underwent subsequent liver resection.

**CASE PRESENTATION:**

A 55-year-old man was referred to our department with a suspected intrahepatic cholangiocarcinoma, based on imaging findings. Anatomic liver resection of segment 8 was performed, and the tumor was confirmed to be HEHE from the pathological findings. Fifteen months later, a solitary recurrence developed in segment 7. After a 5-month observation period, partial liver resection was performed, and the tumor was consistent with recurrent HEHE. The postoperative course was uneventful, and the patient remained recurrence-free for 9 months following the procedure.

**CONCLUSIONS:**

Repeat liver resection may be a feasible treatment option for patients with solitary recurrent HEHE.

## Abbreviations

AFP
: alpha-fetoprotein
CA19-9
: carbohydrate antigen 19-9
CD
: cluster determinant
CE-IOUS
: contrast enhanced intraoperative ultrasound
CEA
: carcinoembryonic antigen
CT
: computed tomography
EOB-MRI
: ethoxybenzyl-diethylene-triaminepentaacetic acid-enhanced MRI
HEHE
: hepatic epithelioid hemangioendothelioma
ICG R15
: indocyanine green retention rate at 15 minutes
MRI
: magnetic resonance imaging
PET-CT
: positron emission tomographycomputed tomography
PIVKA-II
: protein induced by vitamin K absence or antagonist-II
VEGF
: vascular endothelial growth factor
VEGFR
: VEGF receptor

## INTRODUCTION

Hepatic epithelioid hemangioendothelioma (HEHE) is a rare vascular tumor with malignant potential, and has an aggressiveness graded between hemangioma and hemangiosarcoma.^1)^ While most HEHE patients present with diffuse tumors involving both lobes of the liver, a smaller subset has a solitary tumor at diagnosis.^2)^ Although no consensus exists for a standardized treatment strategy because of the rarity of the disease, liver transplantation or radical surgical resection is currently considered as the effective treatments.^3)^ Liver transplantation is a feasible treatment for patients with multiple and unresectable HEHEs.^4)^ Liver resection is generally selected for patients with solitary or a few tumors, despite the risk of aggressive recurrences.^5)^ Previous studies have reported a recurrence rate of 45.7% regardless of the treatment modality, with the liver as the most common recurrence site.^2)^ However, there have been a few case reports in which repeat liver resections were performed for primary and recurrent HEHE. Furthermore, in the medical literature, only 1 case report has been reported in which a patient with solitary primary and recurrent HEHE underwent repeat liver resections.^6)^ Herein, we present the rare case of a patient with solitary primary HEHE, who experienced solitary recurrence following liver resection and underwent subsequent liver resection.

## CASE PRESENTATION

**Fig. 1:**
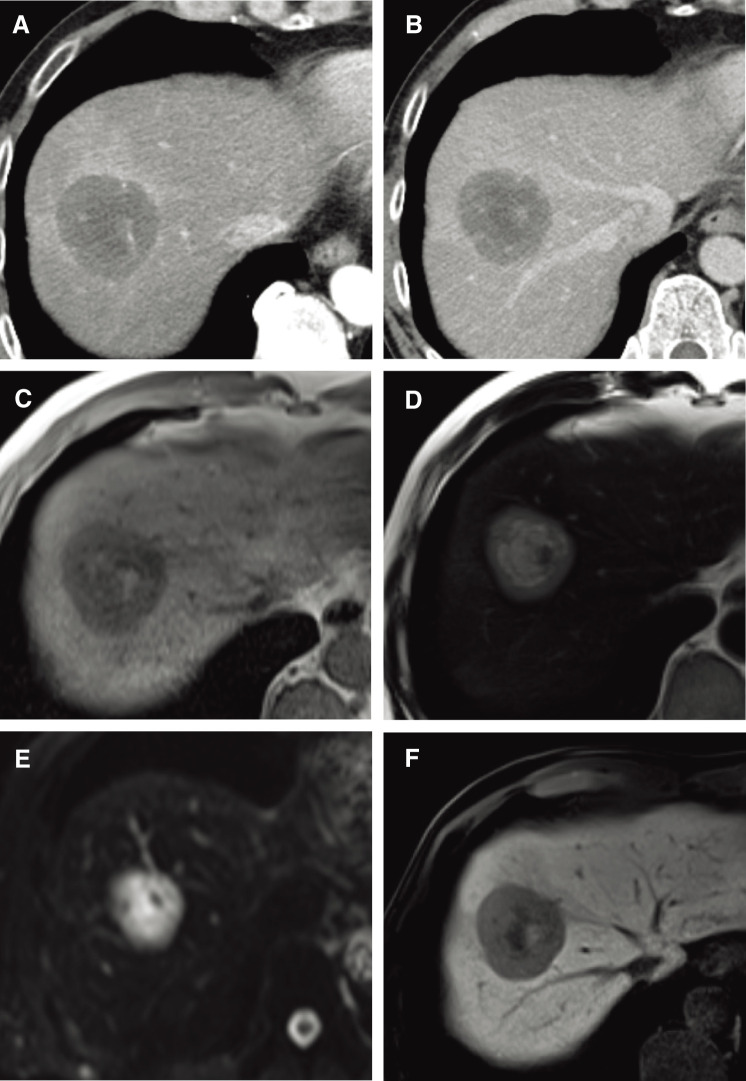
Imaging findings of primary HEHE. (**A**) Arterial phase of CT. (**B**) Portal phase of CT. (**C**) T1-weighted imaging of MRI. (**D**) T2-weighted imaging of MRI. (**E**) Diffusion weighted imaging of MRI. (**F**) Hepatobiliary phase of EOB-MRI. CT, computed tomography; EOB-MRI, ethoxybenzyl-diethylene-triaminepentaacetic acid-enhanced MRI; HEHE, hepatic epithelioid hemangioendothelioma; MRI, magnetic resonance imaging

**Fig. 2:**
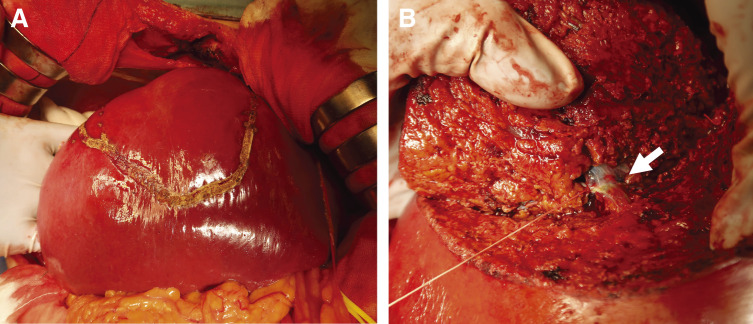
Intraoperative findings of anatomic resection of segment 8 for primary HEHE. (**A**) Transection line of segment 8 on the liver surface. (**B**) Glissonean pedicle of G8 (white arrow). HEHE, hepatic epithelioid hemangioendothelioma

**Fig. 3:**
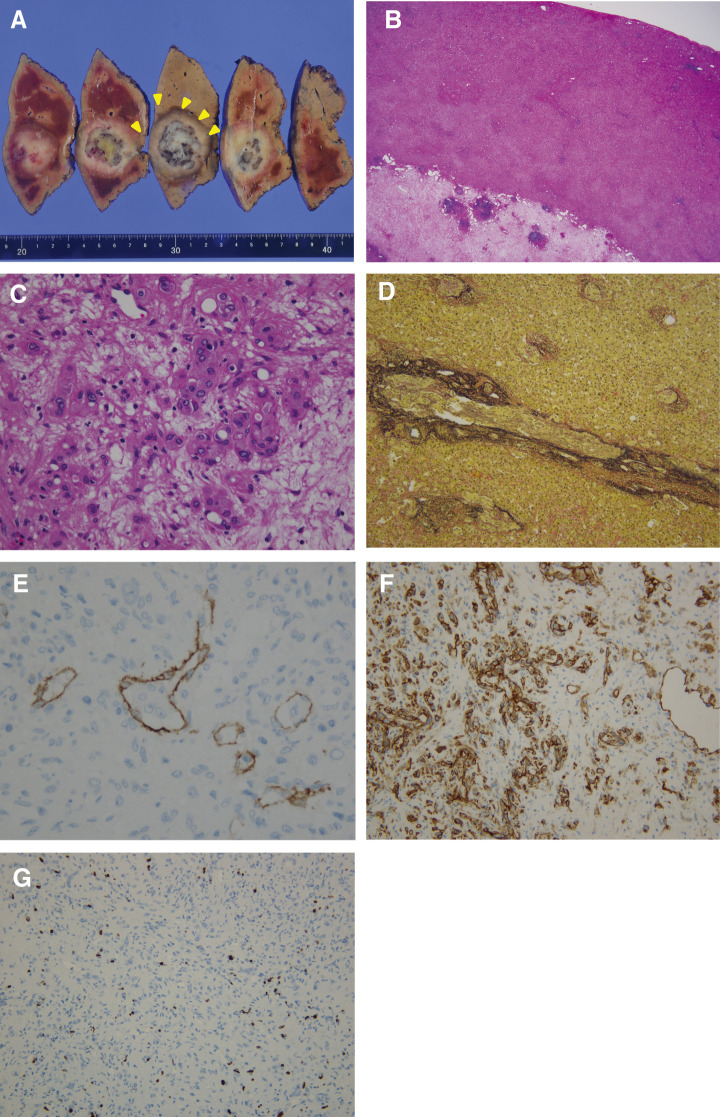
Immunohistopathological findings of primary HEHE. (**A**) Macroscopic findings. The tumor exhibits a 2-layered structure due to differences in cell density, and yellow arrowheads show the outline of the tumor. (**B**) HE staining (×12.5). (**C**) HE staining (×400). (**D**) Vascular invasion image in immunohistochemical staining for CD34 (×400). (**E**) Lymphatic invasion image in immunohistochemical staining for D2-40 (×400). (**F**) Immunohistochemical staining for CD31 (×200). (**G**) Ki-67 labeling index (×200). CD, cluster determinant; HE, hematoxylin-eosin; HEHE, hepatic epithelioid hemangioendothelioma

**Fig. 4:**
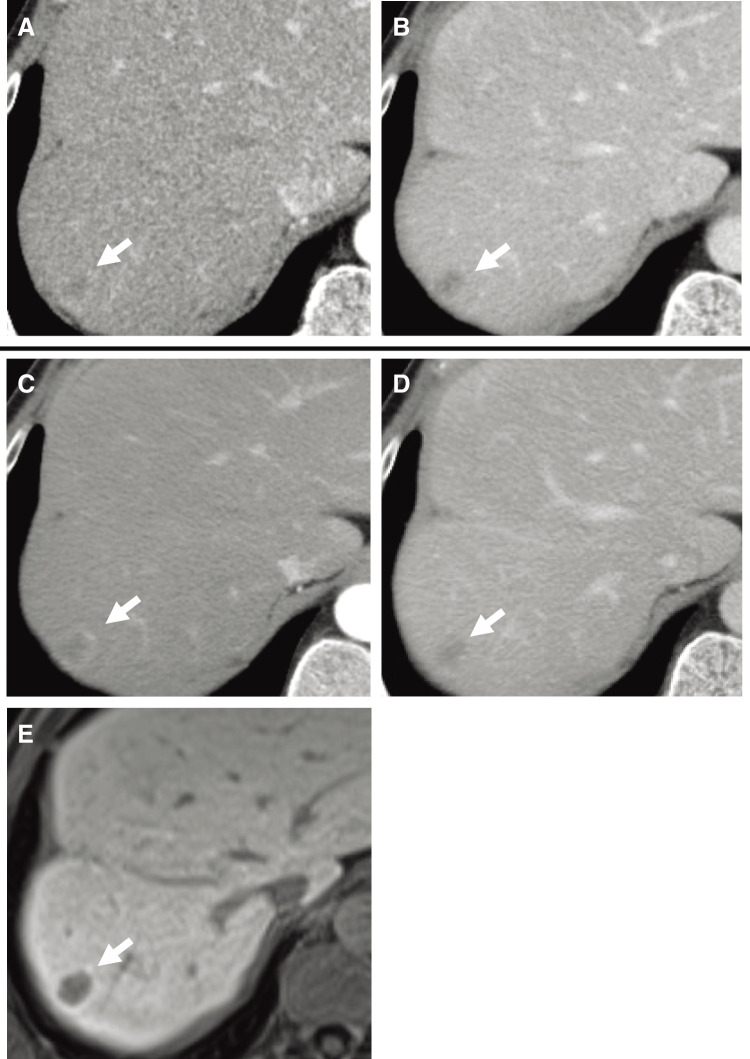
Imaging findings of recurrent HEHE (white arrow). Recurrent HEHE at diagnosis in (**A**) arterial phase and (**B**) portal phase of CT. (**C**) Recurrent HEHE after 5-months’ observation in arterial phase, (**D**) portal phase of CT, and (**E**) hepatobiliary phase of EOB-MRI. CT, computed tomography; EOB-MRI, ethoxybenzyl-diethylene-triaminepentaacetic acid-enhanced MRI; HEHE, hepatic epithelioid hemangioendothelioma; MRI, magnetic resonance imaging

**Fig. 5:**
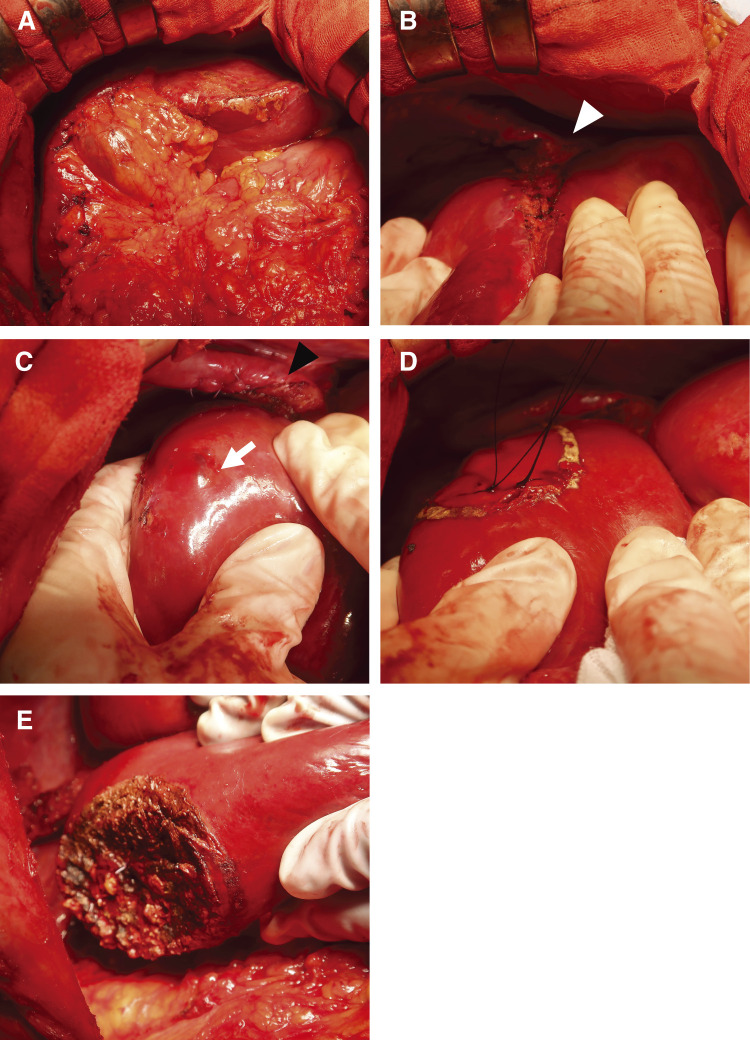
Intraoperative findings of partial liver resection of segment 7 for recurrent HEHE. (**A**) Adhesion around the liver (after adhesiolysis). **(B**) Severe adhesion between the diaphragm and the cut surface of the liver (white arrowhead). (**C**) The recurrent tumor on the liver surface (white arrow). (**D**) Transection line of partial liver resection of segment 7 on the liver surface. (**E**) Cut surface after partial liver resection of segment 7. HEHE, hepatic epithelioid hemangioendothelioma

**Fig. 6:**
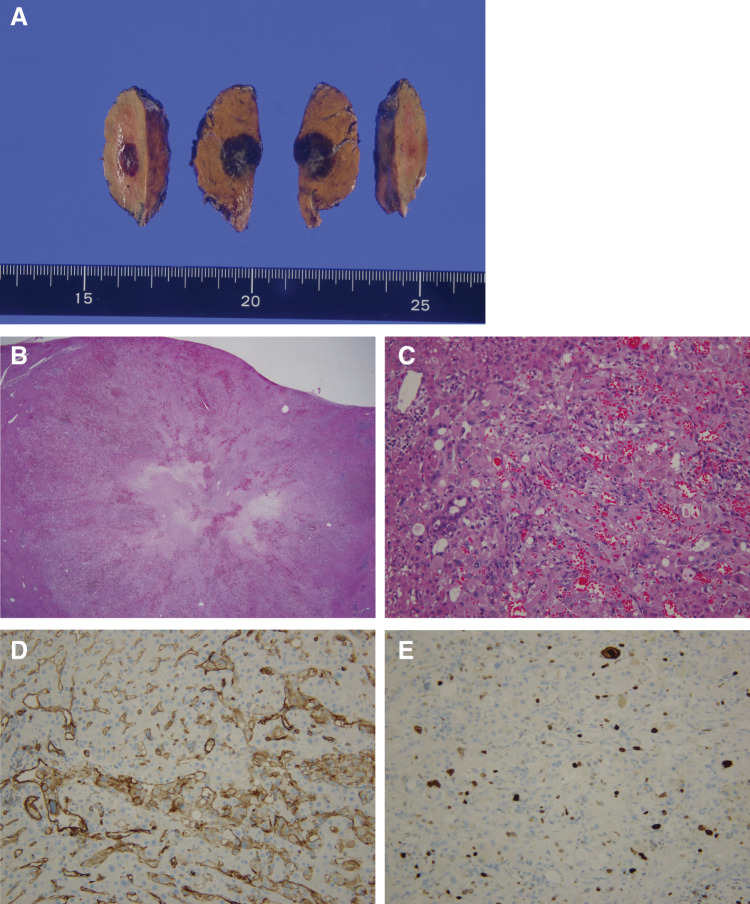
Immunohistopathological findings of recurrent HEHE. (**A**) Macroscopic findings. (**B**) HE staining (×12.5). (**C**) HE staining (×200). (**D**) Immunohistochemical staining for CD31 (×200). (**E**) Ki-67 labeling index (×200). CD, cluster determinant; HE, hematoxylin-eosin; HEHE, hepatic epithelioid hemangioendothelioma

A 55-year-old man with a history of hemorrhoids presented to a local hospital for a medical examination. Abdominal ultrasonography revealed a hypoechoic liver tumor in segment 8, measuring 31 mm in diameter. While contrast-enhanced computed tomography (CT) showed no enhancement in or around the tumor, the slightly elevated CT value raised concerns about potential malignancy. The patient underwent regular ultrasound follow-up, and the tumor increased in size from 31 to 48 mm in diameter over 2 years, prompting referral to our hospital. Contrast-enhanced CT revealed a hypovascular tumor measuring 50 mm at segment 8, located near the root of the Glissonean pedicle. The tumor showed no enhancement in the arterial phase and only slightly delayed enhancement in the portal phase (**Fig. 1A** and **1B**). Magnetic resonance imaging (MRI) demonstrated low intensity in T1-weighted imaging and high intensity in T2-weighted imaging (**Fig. 1C** and **1D**) and high intensity in diffusion weighted imaging (**Fig. 1E**). Gadolinium ethoxybenzyl-diethylene-triaminepentaacetic acid-enhanced MRI (EOB-MRI) showed low intensity compared with the surrounding liver tissue in the hepatobiliary phase (**Fig. 1F**). Positron emission tomography-computed tomography (PET-CT) was not performed. All the serum tumor marker levels, including alpha-fetoprotein (AFP), protein induced by vitamin K absence or antagonist-II (PIVKA-II), carcinoembryonic antigen (CEA), and carbohydrate antigen 19-9 (CA19-9), were within normal limits. The indocyanine green retention rate at 15 minutes (ICG R15) was 17%, and the Child-Pugh score was 5, A. Based on these findings, the patient was diagnosed with suspected intrahepatic cholangiocarcinoma (T2N0M0, Stage II according to the Japanese Society of Hepato-Biliary-Pancreatic Surgery’s sixth edition General Rules for Clinical and Pathological Studies on Cancer of the Biliary Tract) and schedules for open anatomic resection of segment 8. On laparotomy, the tumor was firm and palpable in segment 8. Intraoperative ultrasound revealed a 2-layer structure with a hyperechoic inner layer and a hypoechoic outer layer. Contrast enhanced intraoperative ultrasound (CE-IOUS) demonstrated enhancement in the vascular phase and hypoechoic in the Kupffer phase, and there were no other lesions in the liver. As planned, anatomic liver resection of segment 8 was performed with negative surgical margins (**Fig. 2**). The operation time was 4 hours 33 minutes, with an intraoperative blood loss of 460 mL. Histopathological examination of the tumor suggested a diagnosis of HEHE with severe lymphovascular invasion in the intrahepatic portal veins, hepatic veins, and lymph vessels (**Fig. 3A**–**3E**). Immunohistochemical staining confirmed the neoplastic cells were diffusely positive for cluster determinant (CD)31 (**Fig. 3F**). The Ki-67 labeling index was 5% (**Fig. 3G**). The postoperative course was uneventful, and the patient underwent regular CT follow-up. Fifteen months after surgery, contrast-enhanced CT revealed a hypovascular tumor with ring enhancement, measuring 13 mm in diameter on the liver surface of segment 7, suggesting a possible recurrence of HEHE (**Fig. 4A** and **4B**). After discussing treatment options with the patient, they opted for a watch-and-wait approach. Five months later, contrast-enhanced CT and EOB-MRI showed a slight increase in tumor size to 18 mm but no new lesions (**Fig. 4C**–**4E**). The patient was then diagnosed with solitary recurrent HEHE and agreed to repeat liver resection. The ICG R15 was 6.5%, and the Child–Pugh score remained 5A. Open partial liver resection of segment 7 was performed. Intraoperative findings revealed severe adhesions around the liver, as well as between the diaphragm and the cut surface of the liver (**Fig. 5A** and **5B**). The tumor was located near the surface of the liver in segment 7 (**Fig. 5C**–**5E**), and CE-IOUS showed no enhancement in the vascular phase and hypoechoic in the Kupffer phase. The operation time was 4 hours and 12 minutes, with an intraoperative blood loss of 362 mL. Histopathological examination of the tumor revealed findings similar to the primary tumor, including hemorrhage and hemosiderin deposition. The surgical margin was negative, and lymphovascular invasion remained severe (**Fig. 6A**–**6C**). Immunohistochemical staining showed diffusely positive for CD31 (**Fig. 6D**), and the Ki-67 labeling index was 7% (**Fig. 6E**). The postoperative course was uneventful, and CT scans conducted 9 months after the second liver resection revealed no recurrence of HEHE.

## DISCUSSION

HEHE is a rare hepatic vascular tumor with an incidence of 1–2 per million individuals,^7)^ categorized as malignant potential tumor according to the World Health Organization classification.^8)^ HEHE is also classified as a low to moderate malignant tumor, with the degree of malignancy falling between that of hemangioma and hemangiosarcoma.^1,9)^ The mean age of HEHE patients is approximately 40 years, with a male-to-female ratio of 2:3.^2)^ HEHE presents with nonspecific clinical manifestations, ranging from asymptomatic, right upper abdomen pain, ascites, and weight loss.^3,10)^ Laboratory examinations show abnormal liver function in approximately 40% of the patients with HEHE; however, the levels of tumor markers including AFP, CEA, and CA-19 were mostly normal.^11)^ On imaging, HEHE appears as a low-density tumor on plain CT with minor peripheral enhancement on contrast-enhanced CT. MRI generally shows low intensity in T1-weighted imaging and high intensity in T2-weighted imaging, with slight high intensity in the periphery.^12,13)^ Fluorodeoxyglucose uptake on PET-CT is observed in 66% of HEHE patients.^14)^ Histologically, HEHE is characterized by nests and cords of epithelial-like endothelial cells embedded in a transparent mucus matrix.^15,16)^ Immunohistopathological markers such as Factor VIII-related antigen, CD34, CD31, and vimentin are positive in most HEHE patients.^11)^ Both HEHE and angiosarcoma are known to be positive for endothelial markers, making their differential diagnosis important. In cases where only a portion of the tumor, such as a needle biopsy sample, is available for evaluation, distinguishing between angiosarcoma and HEHE can be challenging. However, in surgical specimens, as in the present case, when fibrous tissue is prominent in the central area, the differentiation between HEHE and angiosarcoma is relatively straightforward. While the etiopathogenic mechanisms remain unclear, recent studies have identified the activation of the vascular endothelial growth factor (VEGF)-VEGF receptor (VEGFR) signaling pathway.^17)^ Most patients with HEHE present with multiple tumors at diagnosis, with only 13% having solitary tumors. Extrahepatic involvement is detected in 36.6% of patients, with common sites being the lung (8.5%), lymph nodes (7.7%), peritoneum (6.1%), and bone (4.9%). The recurrence rate is 45.7% regardless of treatment, and the liver is the most common recurrence site (27.7%).^2)^

Due to the low incidence of HEHE, there is no established gold standard treatment. Several approaches, including liver resection, liver transplantation, chemoradiotherapy, and the watch-and-wait approach, have been reported.^18)^ However, prospective randomized studies comparing these treatments are lacking due to the disease’s rarity. Liver transplantation is considered the best option for unresectable HEHE with multiple or bilobar tumors,^18)^ and limited extrahepatic disease is generally not a contraindication.^19)^ The largest multicenter study showed favorable 5-year overall survival after liver transplantation (79.5%),^19)^ similar to that of liver resection (75.2%).^4)^ However, liver transplantation carries risks of high mortality/morbidity and requires lifelong immunosuppression.^20)^

Several studies have suggested that complete liver resection offers a better prognosis than other treatments.^4,18,21,22)^ However, liver resection tends to be selected in cases with favorable disease condition, which may influence the prognosis. The indication for liver resection for HEHE remains unclear. Giovanardi et al. reported that liver resection should be indicated only when the tumor is solitary or oligonodular and unilobar without the extrahepatic disease.^18)^ Grotz et al. suggested that patients with solitary HEHE smaller than 10 cm should undergo liver resection, while those with solitary tumors larger than 10 cm or multiple tumors should undergo liver transplantation.^23)^ By contrast, Na et al. reported that the number of tumors (more than 4 or less) was not related to recurrence after liver resection.^22)^ The appropriate surgical margin also remains controversial,^24)^ and palliative liver resection is not recommended due to HEHE’s aggressive behavior after resection.^11)^ Laparoscopic liver resection may be suitable for tumors near the liver surface.^24,25)^ In this case, the patient underwent open anatomic liver resection for the primary tumor, initially suspected to be intrahepatic cholangiocarcinoma, and located near the root of the Glissonean pedicle. Even if HEHE was diagnosed preoperatively by biopsy, since liver resection has been reported as a potentially effective treatment for selected cases as described above, the same surgical approach performed in this case would likely have been selected to achieve an adequate surgical margin. For the recurrent tumor, located near the cut surface of the liver with suspected adhesions, open repeat liver resection was selected. The severe adhesions between the diaphragm and the cut surface of the liver were confirmed during the procedure.

Given the high recurrence rate of HEHE, treatment decisions for recurrent disease can be challenging. To date, only a few cases in which repeat liver resections were performed for recurrent HEHE have been reported.^6,22,24)^ Terasaki et al. described a successful case of multiple primary and recurrent HEHEs treated with laparoscopic repeat liver resections.^24)^ Although liver resection is generally not indicated in patients with multiple HEHEs, they were successfully treated for multiple primary and recurrent HEHEs because the tumors were located near the surface of the liver. Additionally, adhesion around the liver was not severe, since the first liver resection was performed by laparoscopic approach. To our knowledge, only 1 other case report exists of a patient undergoing repeat liver resections for solitary primary and recurrent HEHE, similar to our presented case.^26)^ In that case, the patient developed recurrent HEHE 10 years after the primary resection and survived for 17 years following the second resection. These findings emphasize the importance of long-term postoperative surveillance and suggest repeat liver resection as a potential treatment strategy for selected cases of recurrent HEHE. However, surgical indication must be carefully considered, as aggressive tumor progression has been reported following liver resection for HEHE.^5)^

## CONCLUSIONS

We presented an extremely rare case of a patient with solitary primary and recurrent HEHE who underwent successful repeat liver resections. While liver resection may be an optimal treatment option for selected patients with solitary recurrent HEHE, careful consideration of surgical indication is essential.

## ACKNOWLEDGMENTS

The authors sincerely thank Editage (www.editage.jp) for the English language review.

## DECLARATIONS

### Funding

This study was partly supported by the Grant for the National Centre for Global Health and Medicine (Grant No. B2041105).

### Authors’ contributions

YY and FI contributed to the study conception and design.

All authors contributed to data acquisition and analysis.

YY and FI were the major contributors to writing the manuscript.

All authors have read and approved the final manuscript.

All authors have agreed to take responsibility for all aspects of the research.

### Availability of data and materials

Data sharing is not applicable to this article as no datasets were generated or analyzed during the current study.

### Ethics approval and consent to participate

This work does not require ethical considerations or approval. Informed consent to participate in this study was obtained from the patient.

### Consent for publication

Informed consent for publication of this case report was obtained from the patient.

### Competing interests

The authors declare that there are no conflicts of interest.
